# Impact of abdominal obesity on the risk of glioma development in patients with diabetes: A nationwide population-based cohort study in Korea

**DOI:** 10.1371/journal.pone.0283023

**Published:** 2023-03-16

**Authors:** Hyunji Sang, Yun Kyung Cho, Kyungdo Han, Eun Hee Koh

**Affiliations:** 1 Department of Internal Medicine, Asan Medical Center, University of Ulsan College of Medicine, Seoul, Republic of Korea; 2 Asan Diabetes Center, Asan Medical Center, Seoul, Republic of Korea; 3 Department of Statistics and Actuarial Science, Soongsil University, Seoul, Republic of Korea; University of Auckland / University of Oxford, NEW ZEALAND

## Abstract

**Background:**

Abdominal obesity has been suggested as a risk factor for glioma; however, it is unclear whether this association applies to people with diabetes. This study examined the association between abdominal obesity and the risk of developing gliomas in diabetic patients.

**Methods:**

We conducted a retrospective cohort study using the National Health Insurance System of South Korea from 2009 to 2012. The primary outcome was the incidence of newly diagnosed gliomas according to waist circumference (WC), and subgroup analyses were performed according to demographic characteristics and diabetes status including disease duration, number of oral hypoglycemic agents, and insulin use.

**Results:**

Of a total of 1,893,057 participants, 1,846 (0.10%) cases of gliomas occurred. After adjusting for confounding factors, WC ≥90 cm (men)/85 cm (women) was associated with significantly higher risks of gliomas (adjusted HR [95% CI]; 1.279 [1.053, 1.554], 1.317 [1.048, 1.655], and 1.369 [1.037, 1.807] in the WC <95 cm (men)/90 cm (women) group, WC <100 cm (men)/95 cm (women) group, and WC ≥100 cm (men)/95 cm (women) group, respectively). Subgroup analysis showed that patients with larger WC had a consistently higher incidence of glioma than their lean counterparts, except for insulin users (insulin user vs. nonuser, *P* for interaction = .03).

**Conclusions:**

Abdominal obesity was associated with the development of gliomas in diabetic patients in a nationwide population-based database. Further study is needed in diabetic patients to stratify the risk for glioma development according to WC and to establish the underlying mechanism of carcinogenesis.

## Introduction

Glioma is the most common primary intracranial tumor, accounting for over 80% of all malignant brain tumors [1]. Glioblastoma is the most common and aggressive subtype among gliomas, with a mean overall survival of 14.6 months and a 2-year survival rate of 26.5% with conventional therapy [2]. So far, only a few well-established risk factors for the development of glioma have been identified [3], including age, male sex, Caucasian ethnicity, and certain uncommon genetic disorders [1, 4]. The only known modifiable risk factor for glioma is the dose of ionizing radiation [5].

Obesity-related factors are increasingly becoming identified as modifiable risk factors for the development of certain malignancies, including breast and colorectal cancers [5]. However, epidemiological observational studies have reported conflicting results on the roles of obesity-related comorbidities in the development of glioma, with only a subset of studies indicating a significant link [6–11]. Recently, Ahn et al. reported that abdominal obesity was a major risk factor for both men and women in terms of glioma [12], which suggests that a simple, measurable indicator such as waist circumference (WC) could be helpful for risk stratification for glioma.

Patients with diabetes have a high prevalence of abdominal obesity [13]. In Korea, the prevalence of abdominal obesity has steadily increased in the general public over the last 11 years from 2009 to 2019 [14], reaching 29.3% in men and 19.0% in women in 2019 [14]. The incidence of abdominal obesity in adults with diabetes is much higher than that in the general public, as 54% of diabetic patients were shown to have abdominal obesity according to the 2020 Diabetes fact sheet in Korea [15]. Therefore, abdominal obesity-related health outcomes in patients with diabetes are a significant matter of concern.

In this study, we aimed to evaluate the association of abdominal obesity with the risk of glioma development in patients with diabetes using a large-scale nationwide population database in Korea. We adapted WC as a parameter of abdominal obesity. We further conducted comprehensive subgroup analyses according to diabetes status, including diabetes duration, number of oral hypoglycemic agents, and insulin use in addition to demographic characteristics and smoking status.

## Methods

### Data source and study population

**Fig 1:**
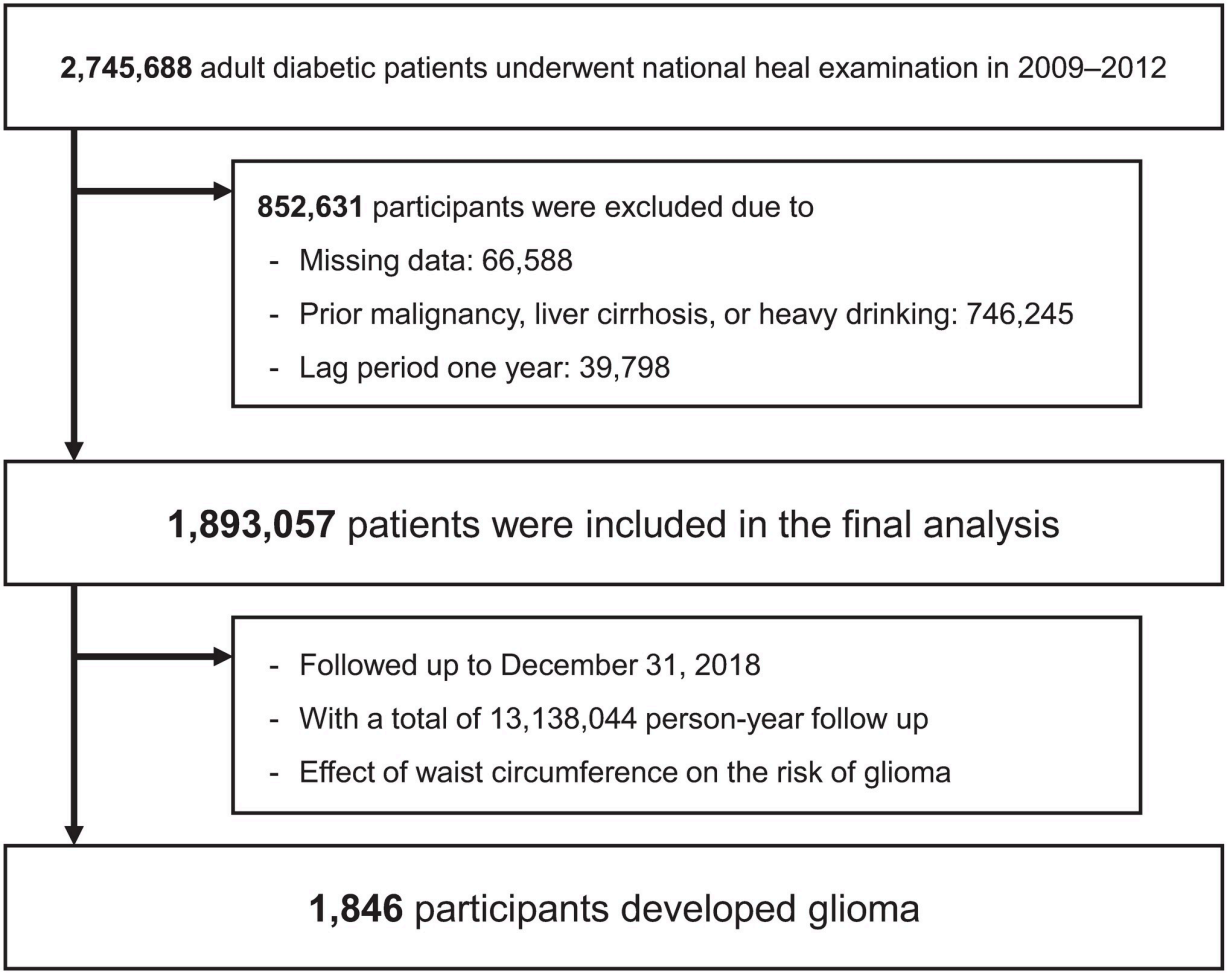
Study flowchart with data from the database of national health insurance service.

In this retrospective cohort study, we used the Korean National Health Insurance System (NHIS) database, which is government-managed and the only insurer providing regular health check-up programs to the public in South Korea. Those enrolled in the health insurance service are recommended to undergo health check-ups at least biennially. In the present study, we included 2,745,688 patients with diabetes aged ≥20 years who underwent a health examination between January 1, 2009 and December 31, 2012. Of them, we excluded 66,588 individuals with missing data and 746,245 individuals with a history of malignancy, including liver cancer or liver cirrhosis, and heavy drinking. Patients who were diagnosed with glioma or died within one year of study enrollment (n = 39,798) were subsequently excluded. Finally, a total of 1,893,057 patients were included in our analyses (Fig 1).

This study was conducted according to the Declaration of Helsinki and approved by the Institutional Review Board of Asan Medical Center (IRB No. 2022–0872). Informed consent was not required because the information used in the analyses was anonymized and de-identified. The reporting of this study followed the Strengthening the Reporting of Observational Studies in Epidemiology (STROBE) reporting guideline for cohort studies.

### Data collection

Comorbidities were mainly defined using a combination of disease history (International Classification of Disease, Tenth Revision [ICD-10] code and self-reported) and medication history for the corresponding disease. Hypertension was defined according to the presence of at least one claim per year under the ICD-10 codes I10–I13 or I15 and at least one claim per year for the prescription of an antihypertensive agent or systolic/diastolic blood pressure ≥140/90 mmHg. Diabetes mellitus was defined according to the following criteria: (1) at least one claim per year under the ICD-10 codes E11–E14 and at least one claim per year for the prescription of antidiabetic medication or (2) fasting glucose level ≥126 mg/dL. Blood samples for the measurement of serum glucose, total cholesterol, triglycerides, high-density lipoprotein cholesterol, and low-density lipoprotein cholesterol levels were drawn after fasting overnight. Body mass index (BMI) was calculated as weight in kilograms divided by height in square meters. The Korean Society for the Study of Obesity recommends using BMI categories of underweight (<18.5 kg/m^2^), normal weight (18.5–22.9 kg/m^2^), overweight (23–24.9 kg/m^2^), obesity (25–29.9 kg/m^2^), and morbid obesity (>30 kg/m^2^) [16, 17]. WC was measured by healthcare providers at the midlevel between the lower ribs and iliac crest using a tape measure in a standing position. Abdominal obesity was defined as a WC ≥90 cm for men and ≥85 cm for women based on the World Health Organization’s recommendations for Asians [17].

### Definition of glioma, outcome variables, and follow-up

The primary outcome was the incidence of newly diagnosed glioma. The ICD-10 code C71 represents intracranial gliomas and includes diffuse gliomas, astrocytic gliomas, glioneuronal and neuronal tumors, and ependymomas [18]. Because all patients with the C71 code received an additional cost coverage service from the NHIS for rare and incurable diseases (i.e., benefit extension policy for rare incurable diseases [BEP]), we defined patients with gliomas as those who were diagnosed with the C71 code and who were registered as having a BEP to ensure more accurate identification of glioma patients [12, 19]. The study population was followed from baseline at the index year to the date of cancer diagnosis or December 31, 2018, whichever occurred first.

### Statistical analysis

For baseline characteristics, continuous variables are presented as mean ± standard deviation, and categorical variables are presented as number (percentage). Baseline data were compared using the chi-squared test to examine relationships between categorical variables and the Student’s *t*-test to compare the mean values of continuous variables. The primary outcome was calculated by dividing the number of incident cases per person-years. Sensitivity analyses were performed to examine the effects of assuming lag periods of 1, 3, and 5 years (S2 and S3 Tables). Kaplan-Meier curve analysis and a log-rank test were performed to analyze the incidence probability of glioma according to the WC categories and the differences among the groups. Hazard ratios (HRs) and 95% confidence intervals (CIs) for glioma development were calculated using a Cox proportional hazards model for each category. In addition, to analyze the difference in the risk of glioma according to the relative difference in WC, the patients were divided into deciles according to their cumulative ranking in WC (D1 to D10) (S1 Table). Statistical analyses were performed using SAS software (v. 9.4; SAS Institute), and a *P* value < .05 was considered significant.

## Results

### Characteristics of the study population

In the study population of 1,893,057 patients, a total of 1,846 (0.10%) cases of glioma developed. Table 1 shows the baseline characteristics of the patients according to the development of glioma. Compared with diabetic patients who did not develop glioma, those who developed glioma were older, had a larger WC, and had a higher prevalence of non-drinkers. Patients who developed glioma also had higher systolic blood pressure and a higher prevalence of those who had diabetes for ≥5 years and those who had ≥3 classes of oral hypoglycemic agents (OHAs).

**Table 1:** Baseline characteristics of the study population.

Characteristics	Development of Glioma, No. (%)		*P* value
	No	Yes	
	1,891,211 (99.9%)	1,846 (0.1%)	
Age, years	57.5 ± 12.6	63.6 ± 10.2	< .001
Male	1,063,595 (56.2)	1,010 (54.7)	.19
Body mass index, kg/m^2^	25.1 ± 3.4	25.1 ± 3.2	.30
Waist circumference, cm	85.1 ± 8.7	86.4 ± 8.3	< .001
Systolic BP, mmHg	128.9 ± 15.9	130.3 ± 15.9	< .001
Diastolic BP, mmHg	78.9 ± 10.2	78.7 ± 10.4	.29
**Lifestyle**			
Current smoker	446,880 (23.6)	418 (22.6)	.42
Alcohol drinking	714,510 (37.8)	575 (31.2)	< .001
Regular physical activity	383,128 (20.3)	394 (21.3)	.25
**Diabetes status**			
Diabetes duration ≥ 5 years	573,002 (30.3)	712 (38.6)	< .001
Insulin use	80,506 (4.3)	95 (5.2)	.06
OHA ≥ 3 classes	126,990 (6.7)	173 (9.4)	< .001
**Laboratory data**			
Fasting blood glucose, mg/dL	144.8 ± 46.8	139.6 ± 42.9	< .001
Total cholesterol, mg/dL	197.8 ± 42.4	194.7 ± 42.5	.001
HDL cholesterol, mg/dL	51.7 ± 21.9	51.0 ± 21.5	.15
LDL cholesterol, mg/dL	113.2 ± 40.7	111.5 ± 42.0	.07
Triglyceride, mg/dL^a^	144.6 (144.4–144.7)	143.3 (139.9–146.8)	.51
Estimated GFR, mL/min/1.73m^2^	84.5 ± 35.7	81.0 ± 24.9	< .001

Data are mean ± standard deviation or n (%) unless indicated otherwise.

^a^ Geometric mean (95% confidence interval).

BP, blood pressure; OHA, oral hypoglycemic agents; HDL, high-density lipoprotein; LDL, low-density lipoprotein; GFR, glomerular filtration rate.

### Risk of incident glioma according to WC categories

**Fig 2:**
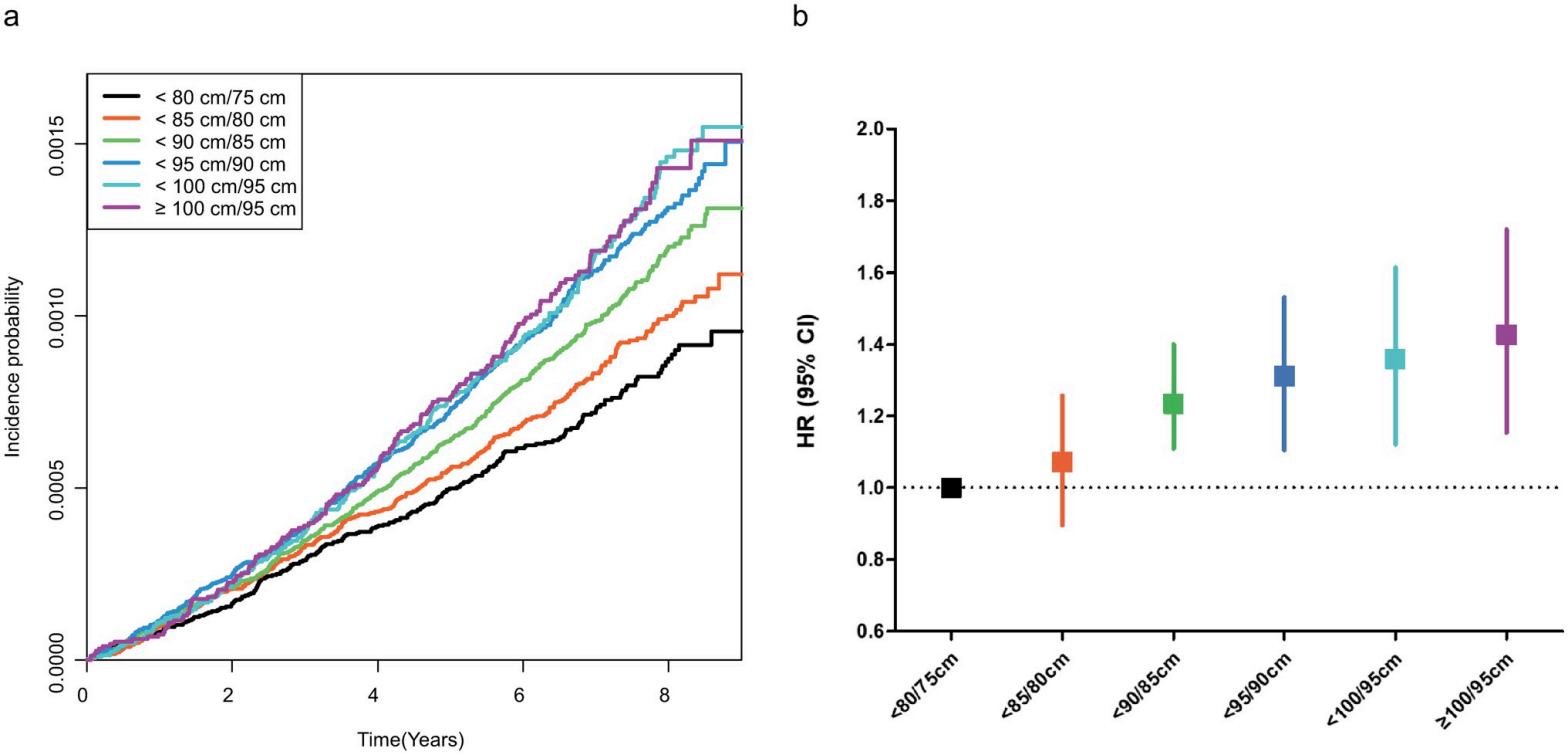
Associations between waist circumference and the risk of glioma. (**a**) Kaplan-Meier estimates of the cumulative incidence of glioma. The analysis was performed in an unadjusted model and the *P* value by log-rank test was < .001 (**b**) Adjusted hazard ratios for glioma according to waist circumference. Hazard ratios were adjusted for age, sex, smoking status, alcohol consumption, household income, body mass index, diabetes duration, number of oral hypoglycemic agents, and insulin use. CI, confidence interval; HR, hazard ratio.

A total of 1,846 cases of glioma occurred during a follow-up period of 13,138,044 person-years. The incidence of glioma was 0.14 per 1,000 person-years in the overall study population. We assessed the association between WC and the risk of incident glioma. When individuals were divided into six groups according to WC, the incidence of glioma was significantly higher in groups with higher WC in the Kaplan-Meier curve analysis (Fig 2a). The incidence rate of glioma proportionally increased as WC increased (Table 2), as patients with WC ≥85 cm (men) or ≥80 cm (women) were at an increased risk of developing gliomas when compared with lean participants (WC <80 cm [men] or <75 cm [women]) (crude HR [95% CI]; 1.350 (1.152, 1.582) in the WC <90 cm (men)/85 cm (women) group; 1.521 [1.292, 1.790] in the WC <95 cm (men)/90 cm (women) group; 1.597 [1.332, 1.916] in the WC <100 cm (men)/95 cm (women) group; 1.602 [1.314, 1.954] in the WC ≥100 cm (men)/95 cm (women) group) (Table 2). Even after adjusting for covariates including age, sex, smoking status, alcohol consumption, household income, BMI, diabetes duration, insulin use, and number of OHAs, the WC ≥90 cm (men)/85 cm (women) groups had significantly higher risks of glioma (adjusted HR [95% CI]; 1.279 [1.053, 1.554] in the WC <95 cm (men)/90 cm (women) group; 1.317 [1.048, 1.655] in the WC <100 cm (men)/95 cm (women) group; 1.369 [1.037, 1.807] in the WC ≥100 cm (men)/95 cm (women) group) (Table 2, Fig 2b). When we analyzed the risk of glioma according to the WC deciles, higher WC was related to an increased risk of glioma (S1 Table).

**Table 2:** Incidence rates and hazard ratios for the development of glioma in diabetic patients according to the waist circumference.

	Total, *n*	Glioma events, *n*	Person-years	Incidence rate/1,000 person-years	HR (95% CI)			
					Model 1	Model 2	Model 3	Model 4
**WC in men/women (cm)**								
< 80/75	312,661	225	2,133,862	0.1015	1 (Reference)	1 (Reference)	1 (Reference)	1 (Reference)
< 85/80	396,907	334	2,761,665	0.1157	1.143 (0.965, 1.354)	1.052 (0.889, 1.246)	1.061 (0.896, 1.256)	1.052 (0.884, 1.253)
< 90/85	470,778	470	3,288,402	0.1111	1.350 (1.152, 1.582)	1.179 (1.006, 1.382)	1.194 (1.018, 1.400)	1.179 (0.989, 1.405)
< 95/90	360,484	405	2,515,986	0.1421	1.521 (1.292, 1.790)	1.282 (1.089, 1.509)	1.301 (1.105, 1.532)	1.279 (1.053, 1.554)
< 100/95	204,324	240	1,420,548	0.1373	1.597 (1.332, 1.916)	1.325 (1.104, 1.590)	1.344 (1.120, 1.614)	1.317 (1.048, 1.655)
≥ 100/95	147,903	172	1,017,580	0.1345	1.602 (1.314, 1.954)	1.390 (1.139, 1.697)	1.409 (1.154, 1.720)	1.369 (1.037, 1.807)

Model 1: unadjusted.

Model 2: adjusted for age and sex.

Model 3: adjusted for age, sex, smoking status, alcohol consumption, and household income.

Model 4: adjusted for age, sex, smoking status, alcohol consumption, household income, body mass index, diabetes duration, insulin use, number of oral hypoglycemic agents.

CI, confidence interval; HR, hazard ratio; WC, waist circumference.

### Subgroup analyses

When we performed a stratified analysis based on age (<65 vs. ≥65 years), sex, smoking status, number of OHA classes (<3 vs. ≥3), diabetes duration (<5 vs. ≥5 years), and insulin use, abdominal obesity was identified as a significant risk factor for the development of glioma in younger individuals (<65 years), men, non-smokers, diabetic patients with longer disease duration, those who are on fewer OHAs classes, and those who are not using insulin (Table 3). Among the clinical factors, insulin use was identified as a significant factor for the association between WC and the risk of glioma (adjusted HR [95% CI]; 0.740 [0.485, 1.130] in insulin users vs. 1.174 [1.043, 1.322] in non-insulin users, *P* for interaction = 0.034) (Table 3).

**Table 3:** Subgroup analyses of the association between waist circumference and glioma in diabetic patients.

Subgroup	WC in men/women (cm)	Total, *n*	Glioma events, *n*	Person-years	Incidence rate/1,000 person-years	Subgroup	
						HR (95% CI)	*P* for interaction
**Age**							
< 65	< 90/85	851,376	545	6,006,756	0.0907	1 (Reference)	.14
	≥ 90/85	458,968	395	3,233,280	0.1222	1.235 (1.063, 1.435)	
**≥** 65	< 90/85	328,970	484	2,177,174	0.2223	1 (Reference)	
	≥ 90/85	253,743	422	1,720,834	0.2452	1.075 (0.928, 1.245)	
**Sex**							
Male	< 90/85	696,313	605	4,803,197	0.1260	1 (Reference)	.77
	≥ 90/85	368,292	405	2,543,088	0.1593	1.162 (1.007, 1.34)	
Female	< 90/85	484,033	424	3,380,733	0.1254	1 (Reference)	
	≥ 90/85	344,419	412	2,411,026	0.1709	1.130 (0.967, 1.321)	
**Currently smoking**							
No	< 90/85	880,745	768	6,127,928	0.1253	1 (Reference)	.93
	≥ 90/85	565,014	660	3,938,629	0.1676	1.150 (1.014, 1.305)	
Yes	< 90/85	299,601	261	2,056,002	0.1270	1 (Reference)	
	≥ 90/85	147,697	157	1,015,485	0.1546	1.139 (0.922, 1.406)	
**BMI ≥25 kg/m** ^**2**^							
No	< 90/85	862,679	776	5,947,812	0.1305	1 (Reference)	.82
	≥ 90/85	113,394	161	772,554.2	0.2071	1.177 (0.988, 1.401)	
Yes	< 90/85	317,667	253	2,236,118	0.1131	1 (Reference)	
	≥ 90/85	599,317	657	4,181,560	0.1571	1.147 (0.985, 1.336)	
**OHAs ≥3 classes**							
No	< 90/85	1,104,112	932	7,651,983	0.1218	1 (Reference)	.73
	≥ 90/85	661,782	741	4,597,214	0.1612	1.154 (1.023, 1.302)	
Yes	< 90/85	76,234	97	531,947	0.1824	1 (Reference)	
	≥ 90/85	50,929	76	356,900	0.2129	1.091 (0.801, 1.485)	
**Diabetes ≥5 years**							
No	< 90/85	827,563	644	5,733,261	0.1123	1 (Reference)	.42
	≥ 90/85	491,780	490	3,419,455	0.1433	1.113 (0.969, 1.278)	
Yes	< 90/85	352,783	385	2,450,669	0.1571	1 (Reference)	
	≥ 90/85	220,931	327	1,534,660	0.2131	1.203 (1.022, 1.416)	
**Insulin use**							
No	< 90/85	1,131,759	969	7,856,354	0.1233	1 (Reference)	.03
	≥ 90/85	680,697	782	4,739,424	0.1650	1.174 (1.043, 1.322)	
Yes	< 90/85	48,587	60	327,576	0.1832	1 (Reference)	
	≥ 90/85	32,014	35	214,690	0.1630	0.740 (0.485, 1.130)	

Hazard ratios were adjusted for age, sex, smoking status, alcohol consumption, household income, body mass index, diabetes duration, number of oral hypoglycemic agents, and insulin use.

BMI, body mass index; CI, confidence interval; HR, hazard ratio; WC, waist circumference; OHA, oral hypoglycemic agents.

## Discussion

By analyzing the records of diabetic patients from a nationwide population-based database, we found that patients with abdominal obesity had a significantly higher risk of developing glioma than lean patients. Considering the higher prevalence of abdominal obesity in diabetic patients than in the general population [12, 13], it is suggested that WC can be used to stratify the risk of glioma in terms of abdominal obesity in diabetic patients as in previous studies. Our results showed that a gradual increase in WC was associated with the risk of glioma compared with patients with standard WC. Furthermore, this positive correlation was confirmed through adjusted regression analysis as well as the incidence probability according to time. These results suggest that increased WC could be used to predict the risk of glioma in diabetic patients.

There has been no clear explanation of the direct interplay between obesity and the development of glioma, especially in patients with diabetes mellitus. Several epidemiologic studies reported that the risk factors of glioma could be different from those of obesity-related tumors [20–22]. The most noteworthy observation was that blood glucose levels seem to be inversely related to the risk of glioma [22]. Although the risk of glioma is associated with abdominal obesity, hyperglycemia (generally thought to be associated with insulin resistance) has a protective effect, unlike in most obesity-related cancers [23]. These previous findings suggest that abdominal obesity and hyperglycemia may have conflicting effects on the risk of glioma [12, 24]. Our study, which exclusively included diabetic patients, showed that the risk of glioma was independently associated with WC in a fully adjusted model, supporting previous reports [4, 11, 12, 25]. Additional proof-of-concept studies are needed to determine the pathogenesis of glioma in comparison with other obesity-related tumors.

To identify the clinically relevant factors for the impact of WC on glioma risk, we performed a subgroup analysis for each clinical variable (Table 3). Patients with larger WC had a consistently higher risk of glioma than their lean counterparts regardless of age, sex, smoking status, BMI, duration of diabetes mellitus, and the number of OHA use. According to the literature, the risk of glioma is higher in the elderly and men [1, 4], and smoking also increases the risk of glioma [19]. Interestingly, in comparison of the HR between patients with high WC (defined as a WC ≥90 cm for men and ≥85 cm for women) and patients with low WC, patients with younger age (<65 years) and currently non-smokers had a slightly higher adjusted HR than their counterparts. In other words, the association of abdominal obesity with glioma was particularly evident in the young population and non-smokers; a possible explanation for this finding is that in the older population and smokers, other factors such as aging and carcinogens included in tobacco may have acted as more powerful determinant factors for the risk of glioma.

In a subgroup analysis according to the status of diabetes, we found that the risk of glioma in patients with high WC was not significantly different according to the duration of diabetes or the number of OHA classes. Instead, the risk for glioma of abdominal obesity was more pronounced in non-insulin users than in insulin users. Diabetes is known to increase the risk of various types of cancer [26–29]; however, in the case of glioma, the risk of glioma paradoxically decreases as the status of diabetes worsens [20, 22, 30, 31]. As higher WC reflects glucose deterioration in diabetic patients [32], insulin use in patients with high WC is likely to reflect poorly controlled diabetes mellitus. So far, it is unclear whether exogenous insulin or insulin secretagogues affect the risk of glioma because other studies showed that exogenous insulin use itself did not significantly affect glioma risk [22]. Additional research is needed to determine whether the occurrence of glioma has a different association with abdominal obesity, hyperglycemia, and insulin-dependent carcinogenesis in comparison with other obesity-related tumors.

There were several limitations to this study. Some data were collected from self-reported questionnaires, which might have been prone to bias. In addition, the differences in histological subtypes were not reflected as we included patients who were registered with the disease code for glioma in the database. Since the susceptibility to abdominal obesity may differ depending on the histological subtype of glioma, further research is needed on this issue. Also, body fat mass was not directly measured and compared due to the limitations of the retrospective study design using a domestic health check-up database. Moreover, the cohort follow-up period of this study was ten years in total, and since a dataset collected for a more extended period could not be obtained, the lag period for analysis was set to 1 year. Nevertheless, sensitivity analyses with lag periods of 3 and 5 years supported the finding that abdominal obesity increased the risk of glioma (S2 and S3 Tables). Finally, our study was not designed to explain how abdominal obesity increases the risk of glioma in diabetic conditions. The underlying mechanism of our findings should be examined in further targeted experimental studies.

## Conclusions

Our nationwide population-based cohort study showed that abdominal obesity was associated with the development of gliomas in diabetic patients. Our results suggest that abdominal obesity management may be beneficial for preventing the development of glioma in diabetic patients. Future research is needed to develop a simple risk stratification model using WC in diabetic patients and to identify the carcinogenic mechanisms of glioma in diabetic patients that differ from other obesity-associated tumors.

## Supporting information

S1 Table Incidence rates and hazard ratios of glioma in diabetic patients according to the deciles of waist circumference. (PDF)

S2 Table Sensitivity analysis: Incidence rates and hazard ratios of glioma in diabetic patients according to the waist circumference in a 3-year lag period. (PDF)

S3 Table Sensitivity analysis: Incidence rates and hazard ratios of glioma in diabetic patients according to the waist circumference in a 5-year lag period. (PDF)
