# Supplementary material for: Impact of abdominal obesity on the risk of glioma development in patients with diabetes: A nationwide population-based cohort study in Korea
Source: PLoS One. 2023 Mar 16;18(3):e0283023. doi: 10.1371/journal.pone.0283023 (PMC10019701; doi:10.1371/journal.pone.0283023)
Supplement: S1 Table — (PDF) [file pone.0283023.s001.pdf]

S1 Table. Incidence rates and hazard ratios of glioma in diabetic patients according to the deciles of waist circumference

	Total, *n*	Glioma events, *n*	Person-Years	Incidence rate/1,000 person-years	HR (95% CI)			
					Model 1	Model 2	Model 3	Model 4
**WC decile**								
D1	176,203	121	1,191,726	0.1015	1 (Reference)	1 (Reference)	1 (Reference)	1 (Reference)
D2	205,178	164	1,417,454	0.1157	1.135 (0.897, 1.436)	1.042 (0.824, 1.318)	1.051 (0.831, 1.329)	1.049 (0.827, 1.331)
D3	194,903	151	1,359,541	0.1111	1.088 (0.857, 1.382)	0.980 (0.772, 1.245)	0.991 (0.780, 1.259)	0.990 (0.775, 1.266)
D4	177,811	176	1,238,354	0.1421	1.393 (1.105, 1.755)	1.204 (0.955, 1.518)	1.221 (0.969, 1.539)	1.219 (0.957, 1.553)
D5	198,785	191	1,391,233	0.1373	1.344 (1.070, 1.688)	1.151 (0.917, 1.446)	1.169 (0.930, 1.468)	1.168 (0.916, 1.489)
D6	177,792	167	1,241,549	0.1345	1.317 (1.043, 1.665)	1.102 (0.872, 1.393)	1.121 (0.887, 1.417)	1.121 (0.871, 1.443)
D7	206,042	217	1,438,287	0.1509	1.478 (1.183, 1.846)	1.206 (0.965, 1.507)	1.227 (0.981, 1.533)	1.227 (0.957, 1.573)
D8	170,671	212	1,192,341	0.1778	1.741 (1.393, 2.177)	1.406 (1.124, 1.759)	1.431 (1.144, 1.790)	1.432 (1.107, 1.851)
D9	187,759	214	1,305,977	0.1639	1.606 (1.285, 2.007)	1.299 (1.039, 1.624)	1.324 (1.059, 1.655)	1.326 (1.016, 1.731)
D10	197,913	233	1, 361,583	0.1711	1.681 (1.350, 2.094)	1.412 (1.133, 1.759)	1.439 (1.154, 1.793)	1.443 (1.075, 1.936)

Model 1: unadjusted
Model 2: adjusted for age and sex
Model 3: adjusted for age, sex, smoking status, alcohol consumption, and household income
Model 4: adjusted for age, sex, smoking status, alcohol consumption, household income, body mass index, diabetes duration, insulin use, number of oral hypoglycemic agents
CI, confidence interval; HR, hazard ratio; WC, waist circumference
