# Supplementary material for: Impact of abdominal obesity on the risk of glioma development in patients with diabetes: A nationwide population-based cohort study in Korea
Source: PLoS One. 2023 Mar 16;18(3):e0283023. doi: 10.1371/journal.pone.0283023 (PMC10019701; doi:10.1371/journal.pone.0283023)
Supplement: S2 Table — (PDF) [file pone.0283023.s002.pdf]

S2 Table. Sensitivity analysis: Incidence rates and hazard ratios of glioma in diabetic patients according to the waist circumference in a 3-year lag period

	Total, *n*	Glioma events, *n*	Person-years	Incidence rate/1,000 person-years	HR (95% CI)			
					**Model 1**	**Model 2**	**Model 3**	**Model 4**
**WC in men/women (cm)**								
< 80/75	305,161	174	1,515,799	0.1148	1 (Reference)	1 (Reference)	1 (Reference)	1 (Reference)
< 85/80	390,401	252	1,973,889	0.1277	1.110 (0.915, 1.346)	1.015 (0.837, 1.232)	1.023 (0.843, 1.241)	1.004 (0.823, 1.226)
< 90/85	463,541	371	2,353,449	0.1576	1.370 (1.144, 1.640)	1.190 (0.993, 1.425)	1.204 (1.005, 1.442)	1.168 (0.957, 1.425)
< 95/90	354,957	317	1,800,021	0.1761	1.530 (1.272, 1.841)	1.283 (1.066, 1.544)	1.301 (1.081, 1.567)	1.248 (1.001, 1.555)
< 100/95	201,076	195	1,014,871	0.1921	1.671 (1.362, 2.049)	1.382 (1.126, 1.697)	1.403 (1.143, 1.723)	1.330 (1.028, 1.720)
≥ 100/95	145,405	139	724,038	0.1920	1.672 (1.338, 2.090)	1.452 (1.161, 1.817)	1.474 (1.177, 1.844)	1.368 (1.001, 1.869)

Model 1: unadjusted
Model 2: adjusted for age and sex
Model 3: adjusted for age, sex, smoking status, alcohol consumption, and household income
Model 4: adjusted for age, sex, smoking status, alcohol consumption, household income, body mass index, diabetes duration, insulin use, number of oral hypoglycemic agents
CI, confidence interval; HR, hazard ratio; WC, waist circumference
