# Supplementary material for: Impact of abdominal obesity on the risk of glioma development in patients with diabetes: A nationwide population-based cohort study in Korea
Source: PLoS One. 2023 Mar 16;18(3):e0283023. doi: 10.1371/journal.pone.0283023 (PMC10019701; doi:10.1371/journal.pone.0283023)
Supplement: S3 Table — (PDF) [file pone.0283023.s003.pdf]

S3 Table. Sensitivity analysis: Incidence rates and hazard ratios of glioma in diabetic patients according to the waist circumference in a 5-year lag period

	Total, *n*	Glioma events, *n*	Person-years	Incidence rate/1,000 person-years	HR (95% CI)			
					Model 1	Model 2	Model 3	Model 4
**WC in men/women (cm)**								
< 80/75	296,819	107	913,700	0.1171	1 (Reference)	1 (Reference)	1 (Reference)	1 (Reference)
< 85/80	381,859	166	1,201,315	0.1382	1.177 (0.923, 1.501)	1.073 (0.842, 1.369)	1.078 (0.845, 1.374)	1.110 (0.864, 1.427)
< 90/85	453,720	244	1,435,783	0.1699	1.448 (1.153, 1.817)	1.251 (0.996, 1.571)	1.260 (1.003, 1.582)	1.328 (1.033, 1.707)
< 95/90	347,304	202	1,097,330	0.1841	1.568 (1.241, 1.982)	1.307 (1.033, 1.653)	1.317 (1.041, 1.666)	1.422 (1.076, 1.880)
< 100/95	196,387	127	617,226	0.2058	1.754 (1.356, 2.268)	1.443 (1.114, 1.868)	1.454 (1.123, 1.882)	1.610 (1.165, 2.226)
≥ 100/95	141,801	91	436,646	0.2084	1.779 (1.346, 2.353)	1.533 (1.158, 2.031)	1.544 (1.165, 2.045)	1.783 (1.206, 2.634)

Model 1: unadjusted
Model 2: adjusted for age and sex
Model 3: adjusted for age, sex, smoking status, alcohol consumption, and household income
Model 4: adjusted for age, sex, smoking status, alcohol consumption, household income, body mass index, diabetes duration, insulin use, number of oral hypoglycemic agents
CI, confidence interval; HR, hazard ratio; WC, waist circumference
